# Acute sympathetic activation blunts the hyperemic and vasodilatory response to passive leg movement

**DOI:** 10.21203/rs.3.rs-4356062/v1

**Published:** 2024-05-10

**Authors:** Brady E Hanson, Joshua F Lee, Ryan S Garten, Zachary Barrett O’Keefe, Gwenael Layec, Bradley A Ruple, D. Walter Wray, Russell S Richardson, Joel D Trinity

**Affiliations:** University of Utah; University of Utah; Virginia Commonwealth University; Mayo Clinic in Rochester; University of Nebraska Omaha; University of Utah; University of Utah; University of Utah; University of Utah

**Keywords:** Vascular function, blood flow regulation, autonomic physiology

## Abstract

Heightened muscle sympathetic nerve activity (MSNA) contributes to impaired vasodilatory capacity and vascular dysfunction associated with aging and cardiovascular disease. The contribution of elevated MSNA to the vasodilatory response during passive leg movement (PLM) has not been adequately addressed. This study sought to test the hypothesis that elevated MSNA diminishes the vasodilatory response to PLM in healthy young males (n = 11, 25 ± 2 year). Post exercise circulatory occlusion (PECO) following 2 min of isometric handgrip (HG) exercise performed at 25% (ExPECO 25%) and 40% (ExPECO 40%) of maximum voluntary contraction was used to incrementally engage the metaboreceptors and augment MSNA. Control trials were performed without PECO (ExCON 25% and ExCON 40%) to account for changes due to HG exercise. PLM was performed 2 min after the cessation of exercise and central and peripheral hemodynamics were assessed. MSNA was directly recorded by microneurography in the peroneal nerve (n = 8). Measures of MSNA (i.e., burst incidences) increased during ExPECO 25% (+ 15 ± 5 burst/100 bpm) and ExPECO 40% (+ 22 ± 4 burst/100 bpm) and returned to pre-HG levels during ExCON trials. Vasodilation, assessed by the change in leg vascular conductance during PLM, was reduced by 16% and 44% during ExPECO 25% and ExPECO 40%, respectively. These findings indicate that elevated MSNA attenuates the vasodilatory response to PLM and that the magnitude of reduction in vasodilation during PLM is graded in relation to the degree of sympathoexcitation.

## INTRODUCTION

The sympathetic nervous system plays a critical role in the short- and long-term regulation of blood pressure and vascular conductance by directly altering vasomotor tone (1–3). Derangements in sympathetic nervous system activity, characterized by heightened muscle sympathetic nerve activity (MSNA), negatively affect vasomotor tone and inhibit vasodilatory capacity. Several cardiovascular diseases including hypertension (4, 5) and heart failure (6, 7), as well as healthy aging (8, 9), are associated with augmented MSNA and concomitant vascular dysfunction. Moreover, both MSNA and vascular function provide important and predictive insight into future adverse cardiovascular events (10, 11). The link between vascular dysfunction and elevated MSNA appears to be more than coincidental, as acute elevations in MSNA result in impaired vascular function as measured by brachial artery flow mediated dilation (FMD) (12–14). Previously, our group superimposed arm exercise on passive leg movement (PLM) and reported reduced PLM-induced vasodilation suggesting that heightened exercise-induced MSNA restricts vasodilation during PLM (15). Clearly, the axis of sympathetic nervous system activity and vascular function is important in the integration and regulation of cardiovascular health. However, how elevated MSNA impacts the vasodilatory response to PLM independent of systemic cardiovascular responses during exercise is not fully understood.

We have previously used PLM to non-invasively assess vascular function (16). PLM provides a reductionist approach to assess movement-induced hyperemia and vasodilation without the confounding increase in oxygen demand and metabolic vasodilation (17, 18). Nearly 80% of the hyperemic and vasodilatory response to PLM is nitric oxide (NO) dependent signifying the utility of this method to provide a valid assessment of NO-mediated vascular endothelial function (16, 19, 20). Moreover, the hyperemic and vasodilatory responses to PLM are significantly reduced by aging, heart failure, and spinal cord injury indicating that PLM provides a sensitive assessment of vascular function across a range of conditions and groups (21–24). As previously described, acute and chronic elevations in MSNA are associated with reductions in vascular function (13, 15, 25). Using a pharmacological approach, we recently reported that modulating α-adrenergic tone directly influences the PLM response (26). Pharmacological approaches provide valuable mechanistic insight but may override the natural physiological state associated with elevated MSNA.

Therefore, this study sought to determine how acute alterations in MSNA impact the vasodilatory and hyperemic responses to PLM. To this end, post exercise circulatory occlusion (PECO) was used following static isometric handgrip exercise at two exercise intensities to engage the metaboreflex and incrementally augment MSNA during PLM (27, 28). We directly tested the hypothesis that graded increases in MSNA would progressively reduce the vasodilatory response to PLM revealing a critical role of sympathetic control in the PLM-induced vasodilatory response.

## METHODS

### Subjects

Eleven healthy males (25 ± 2 y, 178 ± 2 cm, 72 ± 3 kg) volunteered to participate in this research study. Subjects were not taking any prescription medications and were free from overt cardiovascular disease. Protocol approval and written informed consent were obtained according to the University of Utah and Salt Lake City Veteran’s Administration Medical Center (VAMC) Institutional Review Boards, in accordance with the principles outlined in the *Declaration of Helsinki*. All data collection took place at the Salt Lake City VAMC Geriatric Research, Education, and Clinical Center in the Utah Vascular Research Laboratory.

### Experimental Protocol

**Figure 1:**
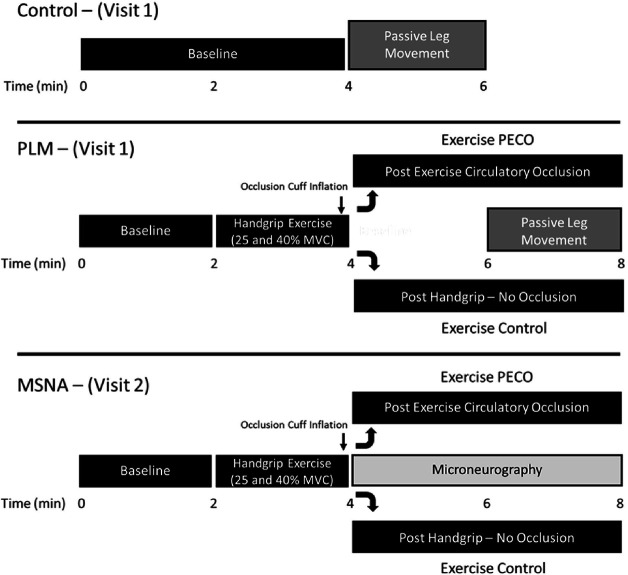
Experimental protocol consisting of two study visits. Visit 1: five passive leg movement (PLM) trials including 1) CONTROL; PLM performed following 4 min of baseline, 2) ExCON 25%; PLM performed after 2 min of static isometric handgrip (HG) exercise at 25% MVC, 3) ExCON 40%; PLM performed after 2 min of static isometric HG exercise at 40% MVC, 4) ExPECO 25%; PLM performed after 2 min of static isometric HG exercise at 25% MVC with concomitant post exercise circulatory occlusion (PECO), and 5) ExPECO 40%; PLM performed after 2 min of static isometric HG exercise at 40% MVC with concomitant PECO. Visit 2: All trials, ExCON 25%, ExCON 40%, ExPECO 25%, and ExPECO 40% were performed followed by muscle sympathetic nerve activity (MSNA) without PLM, and recovery was reduced to 10 min between trials.

Subjects reported to the laboratory following an overnight fast on two separate occasions. The experimental protocol was separated into two visits; one visit focusing on the assessment of central and peripheral hemodynamics during static isometric handgrip (HG) exercise and subsequent PLM (as described below) with and without PECO, while the second visit focused on recording MSNA following HG exercise and PECO without PLM (Fig. 1). We chose to perform two study visits due to the extended duration of the PLM visit (3 to 4 hours) and the difficulty in maintaining an adequate MSNA signal during the PLM assessments. Successful recordings of MSNA were obtained in 8 of the 11 subjects.

Upon arrival at the laboratory, body mass and height were recorded and subjects were instrumented while in the seated upright position. Following instrumentation, subjects performed a series of 3 maximal voluntary handgrip contractions (MVC; 47 ± 3 kg) separated by 1 min recovery. Following 10 min of recovery and attainment of stable central and hemodynamic measures the experimental trials commenced. During the PLM visit (visit 1), a total of 5 experimental trials were performed as depicted in the protocol timeline (Fig. 1). Subjects completed a single control PLM with no intervention and 4 PLMs following the HG intervention. Each trial included 2 min of baseline followed by 2 min of static isometric HG exercise at 25% or 40% MVC. Immediately prior to the end of the 2 min HG exercise an occlusion cuff placed proximal to the elbow was inflated to suprasystolic pressure (250 mmHg) (ExPECO) or left deflated for control trials (ExCON). PECO isolates the contribution of the metaboreceptors to the exercise pressor reflex resulting in elevated mean arterial pressure (MAP) and MSNA during cuff occlusion (28, 29). Two min into the ExPECO or ExCON condition, PLM commenced following established guidelines (30). Briefly, while subjects sat in the upright position with the right leg fully extended (i.e., 180°), a member of the research team moved the leg through a 90° range of motion (i.e., flexion and extension) repeatedly at a rate of 1Hz for 2 min. Subjects were instructed to remain passive and resist the urge to assist with leg movement. A 20 min period of recovery separated trials ensuring stable central and peripheral hemodynamics before the start of the next trial. The order of the trials was balanced.

During the MSNA visit (visit 2) subjects were positioned in the upright seated position and MSNA was continuously recorded from the peroneal nerve. The protocol described above, including handgrip exercise, was repeated, however peripheral hemodynamics were not assessed and PLM was not performed. Additionally, due to concern with maintaining an adequate MSNA signal the recovery between trials was reduced to 10 min. Prior to the start of each trial, MAP was assessed to ensure similar levels between trials.

### Experimental Measurements:

#### Leg blood flow

Common femoral artery blood velocity and vessel diameter were measured in the passively moved limb distal to the inguinal ligament and proximal to the deep and superficial femoral bifurcation with a Logic 7 Doppler ultrasound system (General Electric Medical Systems, Milwaukee, WI, USA) according to published guidelines (31). The ultrasound system was equipped with a linear transducer operating at an imaging frequency of 10 MHz. Blood velocity was measured using the same transducers with a frequency of 5 MHz. All blood velocity measurements were obtained with the probes appropriately positioned to maintain an insonation angle of no more than 60°. Arterial diameter was measured, and mean velocity (*V*_*mean*_) (angle corrected, and intensity-weighted area under the curve) was automatically calculated (Logic 7). Using arterial diameter and *V*_*mean*_, leg blood flow (LBF) in the femoral artery was calculated as *V*_*mean*_ × *π* × (vessel diameter/2)^2^ × 60, and reported as ml·min^−1^. Leg vascular conductance (LVC), an index of vasodilation, was calculated as LBF·MAP^−1^ and expressed as ml·min^−1^ mmHg^−1^.

##### Central hemodynamics:

Heart rate was monitored using a 3 lead electrocardiogram (Biopac). Beat-by-beat changes in stroke volume (SV), cardiac output (CO), and mean arterial pressure (MAP) were continuously determined by a finger photoplethysmography (Finometer: Finapres Medical Systems, Amsterdam, the Netherlands). Additionally, systolic blood pressure (SBP) and diastolic blood pressure (DBP) were determined by auscultation of the left arm with use of an automated sphygmomanometer (Tango^+^, Suntech, Morrisville, NC) to verify the Finometer readings at min 1, 3, 5, and 7 of each experimental trial. MAP was calculated as DBP + 1/3(SBP - DBP).

##### MSNA:

**Figure 2:**
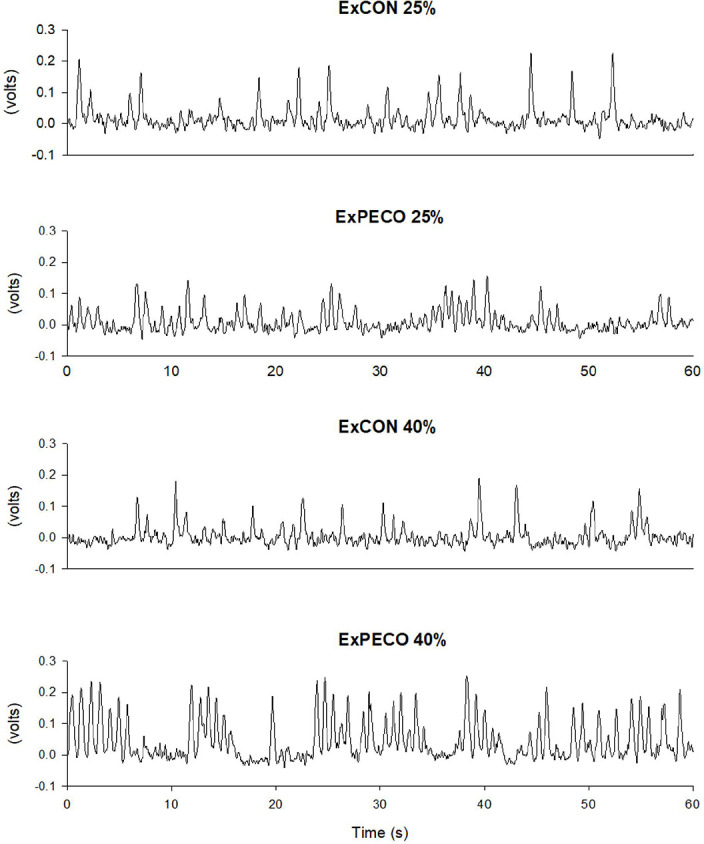
Muscle sympathetic nerve activity (MSNA) following handgrip exercise performed with (ExPECO 25%, ExPECO 40%) and without (ExCON 25%, ExCON 40%) post exercise circulatory occlusion (PECO) in a representative subject.

Multiunit postganglionic MSNA was recorded using standard microneurographic techniques as previously described (32). Following careful mapping of the peroneal nerve near the fibular head on the right leg, a unipolar tungsten microelectrode was inserted into the muscle fascicle. Neural signals were processed by a preamplifier (Nerve traffic analyzer model 662C-3, Iowa Bioengineering, Iowa City, IA, USA) with a total gain of 90,000. Amplified signals were filtered (bandwidth 700–2,000 Hz), rectified, and integrated (time constant 0.1 s) to obtain mean voltage neurograms. Representative tracing of MSNA during ExPECO and ExCON is presented in Fig. 2. Pulse-synchronous MSNA bursts were identified manually by an experienced microneurographer according to appearance (3:1 ratio above the background noise) and timing in relation to the preceding ECG R-wave. Burst frequency (burst·min^−1^) and burst incidence (burst·100 beats^−1^) are reported.

#### Data acquisition

Throughout each protocol, HR, SV, CO, MAP and ECG signals underwent analog-to-digital conversion and were simultaneously acquired (200 Hz) using a data acquisition system, (AcqKnowledge; Biopac Systems, Goleta, CA, USA).

#### Data and statistical analysis

The data acquisition software allowed second-by-second analyses of HR, SV, CO and MAP. All analyses were performed using a 5 s moving average. Second-by-second blood velocities were analyzed on the ultrasound system (GE Logic 7) for the first 60 s of movement, and 12 s averages were assessed from 60 to 120 s of movement. Outcomes related to PLM including peak, delta (Δ) peak, and total (area under the curve, AUC) FBF and FVC, as well as hemodynamic measures and MSNA were assessed via two-way and one-way repeated-measures analysis of variance to determine significant differences between conditions. When a significant main effect (interaction of treatment by time) was observed, further *post hoc* analysis (Holm-Sidak) was performed to determine significant differences between conditions. Significance was set a priori at *α* level of < 0.05, and data are presented as means ± SD. Error bars have been omitted from select figures to improve clarity.

## RESULTS

### Central and peripheral hemodynamics:

#### PRE exercise

**Figure 3:**
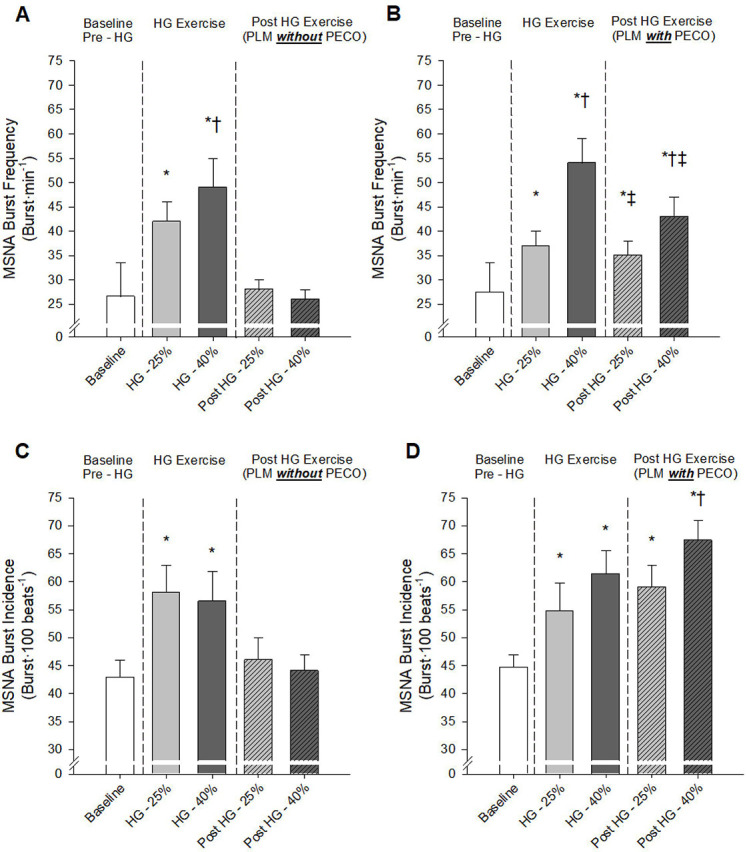
A) Muscle sympathetic nerve activity (MSNA) burst incidence (burst/100 beats) prior to, during, and post handgrip exercise *without* post exercise circulatory occlusion (PECO) and B) *with*PECO and C) MSNA burst frequency (burst/min) *without* PECO and D) *with* PECO. * Significant difference from CONTROL baseline, † significant difference between exercise intensities, ‡ significant difference from same exercise intensity during handgrip exercise, p < 0.05.

Prior to HG exercise, baseline central hemodynamics (HR, SV, and CO) were generally similar between conditions with the only exception of a slight (~ 3 bpm) elevation in HR prior to ExCON 40% and ExPECO 40% when compared to CONTROL (Table 1). MAP was slightly higher at baseline for ExCON 40% than ExCON 25%. There were no baseline differences in HR, SV, CO, or MAP when comparing ExCON 25% to ExPECO 25% or ExCON 40% to ExPECO 40%. Baseline LBF and LVC were not different between any of the conditions (Table 1). Measures of MSNA burst frequency and burst incidence, were not different between conditions (Fig. 3)

#### During exercise

Isometric HG exercise at 25% and 40% of MVC evoked graded elevations in HR, CO, MAP (Table 1), and MSNA burst frequency (Fig. 3A and 3B). MSNA burst incidence was elevated during HG exercise with no differences between ExPECO and ExCON conditions (Fig. 3C and 3D). LBF was augmented by HG exercise, however, no differences were present between conditions. LVC remained unchanged during HG exercise (Table 1).

#### Post exercise

Following HG exercise, HR and CO returned to pre-HG levels while SV was uniformly increased across conditions (Table 1). HR was elevated during ExPECO 40% compared to all other conditions. MAP and MSNA returned to pre-HG levels after cessation of HG exercise during ExCON 25% and ExCON 40% (Tables 1, 2, and Fig. 3) indicating that sympathetic activation returned to baseline levels. Conversely, engaging the metaboreflex after HG exercise resulted in elevated MAP and MSNA during ExPECO 25% and ExPECO 40% (Tables 1, 2, and Fig. 3). Importantly, the metaboreflex-induced increases in MAP and MSNA were graded with respect to the intensity of the preceding HG exercise (ie; ExPECO 40% > ExPECO 25% > CONTROL). Following HG exercise, LBF returned to pre-HG levels during ExCON 25% and ExCON 40% and remained elevated during both ExPECO 25% and ExPECO 40%. LVC remained unchanged following HG exercise.

### Central and peripheral hemodynamics during PLM:

**Figure 4:**
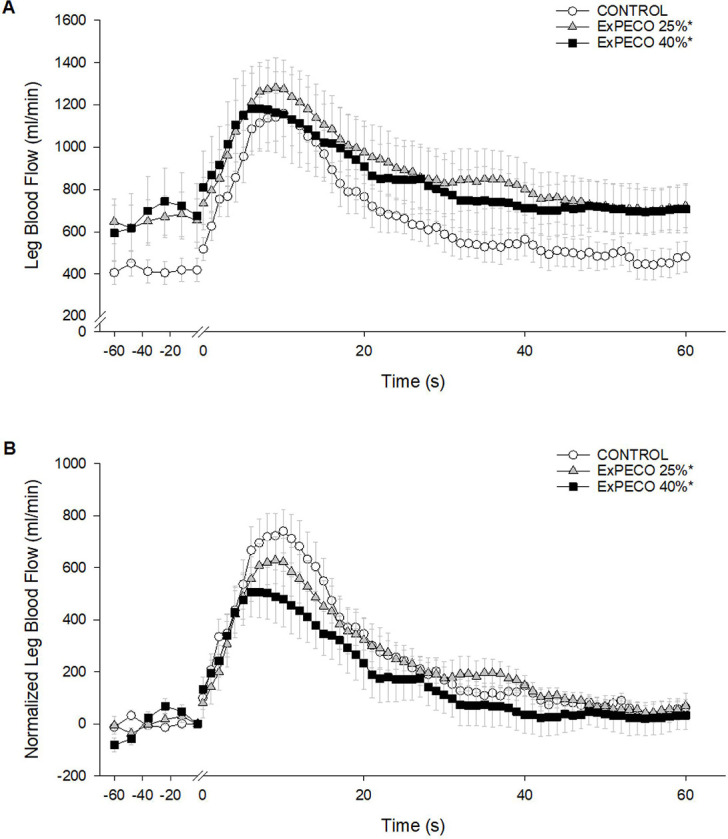
A) Absolute leg blood flow (LBF, ml/min) and B) normalized LBF (baseline normalized to 0, ml/min) during passive leg movement with PECO. Passive leg movement started at time 0 and was performed continuously for 60 s. * Significant difference from CONTROL, p < 0.05.

**Figure 5:**
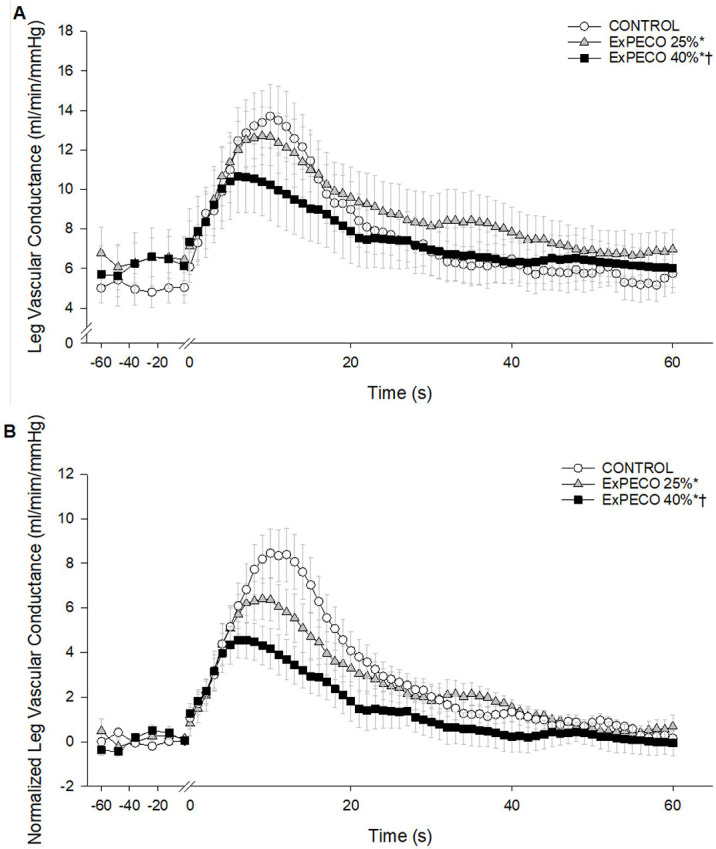
A) Absolute leg vascular conductance (LVC, ml/min/mmHg) and B) normalized LVC (baseline normalized to 0, ml/min/mmHg) during passive leg movement with PECO. Passive leg movement started at time 0 and was performed continuously for 60 s. * Significant difference from CONTROL, † significant difference from PECO 25%, p < 0.05.

PLM-induced a robust and transient increase in LBF (Fig. 4A) and LVC (Fig. 5A). During CONTROL, LBF and LVC increased nearly 3-fold. Significant differences existed between PLM-induced LBF when comparing CONTROL to ExPECO 25% and ExPECO 40% (Fig. 4A). These differences are largely accounted for by the augmented baseline LBF prior to PLM (Table 3 and Fig. 4A). Normalizing for the elevated baseline LBF revealed that the hyperemic response was unaltered by ExPECO 25% but reduced during ExPECO 40% (Fig. 4B).

**Figure 6:**
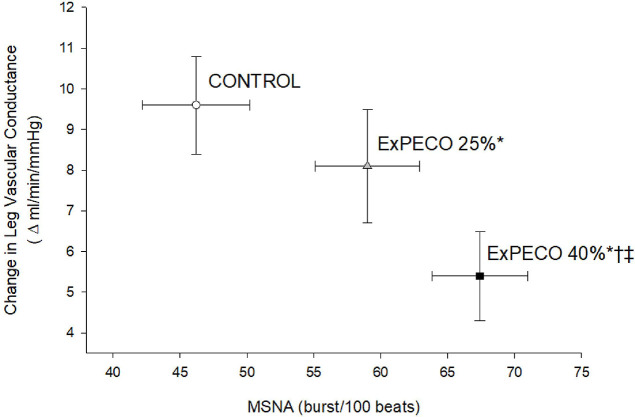
PLM-induced change in LVC (Δ ml/min/mmHg) verse MSNA (burst/100 beats). Graded increases in MSNA evoked by PECO and corresponding reductions in the change in LVC. * Significant difference in MSNA from CONTROL, † significant difference in MSNA from ExPECO 25%, ‡ significant difference in LVC from CONTROL and ExPECO 25%, p < 0.05.

**Figure 7:**
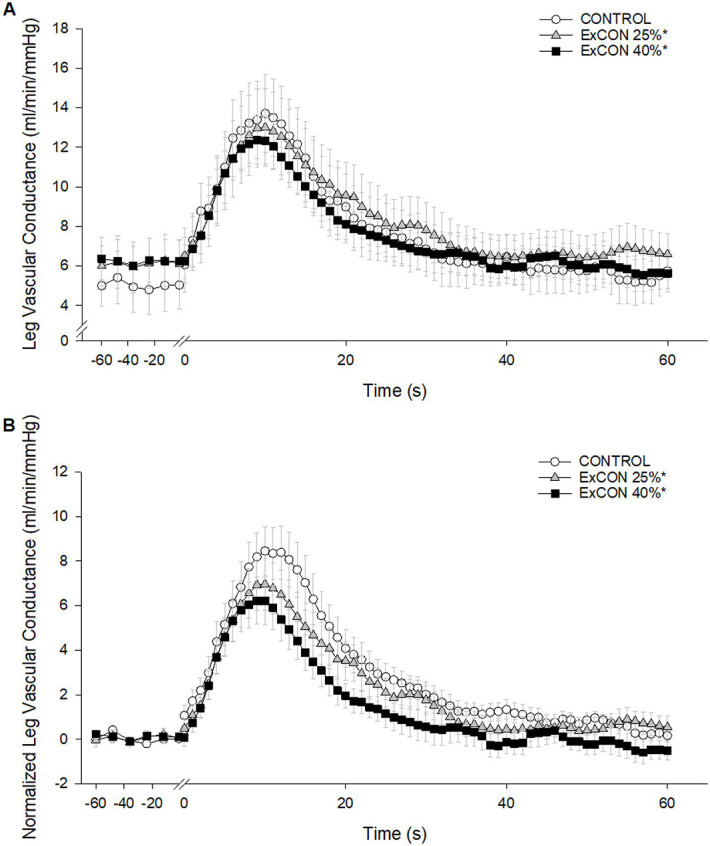
Absolute leg vascular conductance (LVC, ml/min/mmHg) and B) normalized LVC (baseline normalized to 0, ml/min/mmHg) during passive leg movement without PECO. Passive leg movement started at time 0 and was performed continuously for 60 s. * Significant difference from CONTROL, p < 0.05.

During ExPECO 25% and ExPECO 40% the vasodilatory response to PLM was incrementally reduced compared to CONTROL (Figs. 5 and 6). Interestingly, when PLM was preceded by HG exercise alone, a modest, albeit significant reduction in the vasodilatory response to PLM was observed during both ExCON 25% and ExCON 40% (Fig. 7). Unlike the PECO conditions, this reduction in the vasodilatory response was not dependent on the intensity of the preceding HG exercise bout as the reduction in LVC during ExCON 25% and ExCON 40% was similar. Accounting for this unexpected impact of HG on the PLM response revealed that the PLM-induced vasodilatory response was not different between ExCON 25% and ExPECO 25% but was reduced during ExPECO 40% when compared to all other conditions. Additionally, peak and total LVC during ExPECO 40% was attenuated compared to CONTROL and ExPECO 25% (Table 2).

PLM evoked increases in HR, SV, and CO that were not different between conditions (Table 3). Peak HR and SV were not different between conditions while peak CO during ExPECO 40% was higher than CONTROL (Table 2).

## DISCUSSION

Vascular dysfunction and impaired vasodilation often occur in parallel with heightened MSNA, which is associated with aging and the development and progression of cardiovascular disease. However, how acute sympathoexcitation impacts the vasodilatory response to PLM, an assessment of vascular function, has not been determined independent of systemic cardiovascular responses that occur during exercise (15). The novel findings from this study indicate that acute sympathetic activation, accomplished by post exercise circulatory occlusion (PECO), diminishes the hyperemic and vasodilatory responses to PLM, suggesting a regulatory influence of sympathetic nervous system activation on microvascular function in young, healthy males.

### Impact of acute sympathoexcitation on the vasodilatory response to PLM

PLM provides a primarily NO-dependent assessment of vascular function (16, 19, 20). Conditions associated with vascular dysfunction including aging, heart failure, and spinal cord injury exhibit reductions in PLM-induced vasodilation (21, 22). In the current study, acute elevations in MSNA directly impaired the vasodilatory response to PLM. Specifically, during metaboreceptor activation following HG exercise at 25% and 40% MVC (i.e., ExPECO 25% and ExPECO 40%) the change in LVC was reduced by 16% and 44%, respectively, when compared to CONTROL (Fig. 5 and Table 3). This reduction in the change in LVC coincided with significant increases in MSNA (Fig. 6). These findings support recent findings from our group of stepwise reductions in PLM-induced vasodilation during progressive exercise-mediated increases in MSNA (15) and extend these findings to local activation of this reflex loop by the metaboreceptors. The concomitant elevation in MSNA and reduction in the hyperemic response to PLM helps to elucidate factors, in addition to diminished NO bioavailability, that may contribute to previous reports of reduced PLM-induced vasodilation with aging and heart disease (22, 33, 34).

The mechanisms contributing to the reduced vasodilation during elevated sympathetic activation are not entirely clear. During PECO, MSNA increases systemically in response to localized metaboreceptor activation (35). This global increase in sympathetic outflow and stimulus for vasoconstriction may directly oppose the vasodilatory response to PLM by some general, yet unrecognized, mechanism. Conversely, elevated sympathetic outflow may directly inhibit NO. Hijmerring et al. (13) reported a reduction in FMD following acute sympathetic activation (via lower body negative pressure) and attributed the reduction in FMD to a specific inhibitory effect of sympathetic activation on shear-mediated NO release. In vitro evidence suggests that norepinephrine may lead to inactivation of NO (36) lending support to this potential mechanism, however, evidence of this occurring in vivo is lacking (37). A third potential mechanism may involve an imbalance between NO bioavailability and MSNA. Physiologically, NO and MSNA are antagonistic, as NO promotes vasodilation whereas increased MSNA evokes vasoconstriction. Therefore, increasing MSNA without an increase in NO (as would be expected given the peak hyperemic response was not altered by PECO (Table 3)), would shift the balance toward vasoconstriction and reduction in LVC during PLM. Both animal and human models demonstrate an interaction between NO and α-adrenergic function such that NO acts to attenuate α-adrenergic vasoconstriction (38, 39), however, how such a mechanism may manifest in humans during PLM is not certain. Based on the current findings we are unable to determine if the reduction in LVC during PLM is due to a general increase in sympathetically-mediated vasoconstriction, a direct impact on NO, or a combination of these aforementioned mechanisms.

Handgrip exercise without subsequent PECO and heightened MSNA yielded unexpected reductions in the vasodilatory response to PLM ( ExCON 25% and ExCON 40%, Fig. 7). At the cessation of HG exercise, MSNA and MAP returned to pre-HG levels indicating that sympathetic activation likely does not account for the observed reductions in LVC (Fig. 3). Alternatively, the reduced LVC during PLM may be explained by the increase in LBF during HG exercise, which may have resulted in activation of endothelial nitric oxide synthase (eNOS) and a subsequent increase in NO bioavailability (40). Increasing LBF prior to PLM with heating effectively reduced the PLM-induced hyperemic response (41). Recent human and animal work suggests that vascular responsiveness, as measured by vasodilation, is reduced with repeated stimulation due to potential ‘resetting’ of the endothelium (42) or alterations in tissue oxygenation (43). In keeping with this notion, activation of eNOS prior to PLM may have decreased NO bioavailability leading to the observed and marked reduction in LVC at the initiation of PLM. Importantly, this does not negate our finding that MSNA plays a significant role in the LVC response as the increase in LBF during and after HG was nearly identical between ExPECO 25% and ExPECO 40%, yet LVC was reduced during ExPECO 40% when compared to ExPECO 25%.

The impact of acute sympathoexcitation on vascular function, assessed by FMD, has been examined previously during lower body negative pressure, mental stress, cold pressor test, and metaboreceptor engagement (12–14, 44). The findings of these investigations are equivocal, as FMD has been reported to be unchanged, improved, or impaired due to increased sympathetic activity. Reductions in FMD ranging from 40 to 60% have been reported during sympathetic activation induced by lower body negative pressure and cold pressor test (13, 14). Conversely, metaboreceptor activation reportedly increased FMD by nearly 2-fold (12). The variability in the FMD response to acute sympathetic activation in these previous investigations is likely attributed to disparate techniques used to evoke sympathetic activation, as well as uncertainty regarding the magnitude of changes in MSNA. Moreover, uncertainty with regard to the mechanisms contributing to vasodilation during FMD may further confound interpretation of these findings (45, 46). Thus, while differences in methodology between these former studies and the current work preclude a direct comparison, the present findings provide new evidence for the capacity of the sympathetic nervous system to diminish lower limb hyperemic and vasodilatory responsiveness in young, healthy adults.

### Regulation of hyperemia during PLM: impact of acute sympathoexcitation

The unique experimental design employed in the present study provided the opportunity to assess the impact of sympathoexcitation on microvascular reactivity without the influence of metabolic vasodilation that accompanies voluntary exercise (47, 48). This reductionist approach is particularly beneficial considering the complexity of neurovascular control during exercise, when sympathetic vasoconstriction to active skeletal muscle is diminished in direct relation to the exercise intensity, a phenomenon termed functional sympatholysis (49, 50). Thus, the present investigation extends previous investigations examining the impact of augmented MSNA on skeletal muscle blood flow regulation during voluntary exercise (51–54), offering new insight regarding the capacity of sympathetic nervous system activity to regulate muscle blood flow in the resting (inactive) and passively moved limbs.

Prior to PLM, graded activation of the metaboreflex progressively increased MAP, which translated to elevated LBF and unchanged LVC (Table 3) despite a large and systemic increase in sympathetic outflow. This finding, which agrees with several previous reports in humans (54, 55), suggests that a systemic increase in sympathetic outflow does not elicit vasoconstriction in resting, inactive skeletal muscle. Without a vasodilatory stimulus such as PLM, the impact of metaboreceptor activation is unappreciated as LBF is increased yet LVC is unaltered. The full impact of metaboreflex activation and concomitant elevation in sympathetic nervous system activity is only realized after the initiation of PLM to stimulate blood flow. Indeed, PLM evoked a nearly 3-fold increase in hyperemia during CONTROL (Fig. 3). Peak hyperemia remained relatively unchanged despite marked and graded elevations in MSNA and MAP during ExPECO 25% and ExPECO 40% (Table 3). However, the overall vasodilatory response was attenuated during ExPECO 25% and ExPECO 40% when compared to CONTROL (Fig. 5). The reduction in LBF and LVC during PLM provide novel evidence of vasoconstriction even in the ‘active’ (i.e., passively moved leg) muscle during heightened sympathetic activity.

### Central hemodynamic contribution to PLM

The peripheral hemodynamic response to PLM is primarily governed by peripheral factors, however, central factors (HR, SV, and CO) do contribute to alterations reported with body position, age, and cardiovascular disease (18, 21, 22, 56). In the current study HG exercise elicited increases in HR, CO, and MAP. In general, these variables returned to pre-HG levels following termination of HG exercise. By design, the increases in MAP and MSNA remained elevated during ExPECO, albeit at a slightly lower level than HG exercise, confirming the important role of central command in regulating cardiovascular responses to voluntary exercise (54, 57, 58). Despite modest differences in central hemodynamics prior to PLM, the changes induced by PLM were similar between conditions suggesting an important and consistent central hemodynamic contribution to PLM. Overall, the reductions in LBF and LVC during the PECO conditions do not appear to be due to altered central hemodynamics.

### Experimental Considerations

The current findings are novel and significant, however, only male subjects were included. Previous investigations have reported no sex-related differences in the hyperemic and vasodilatory response to PLM (59), thus the current findings may be extrapolated to females as well. Therefore, future investigations including female are warranted to investigate how potential basal differences in MSNA between sex may impact the hyperemic and vasodilatory response to PLM as young males express greater MSNA compared to their female counterparts (60, 61). Additionally, the lack of MSNA measures during the PLM assessment, due to the difficulty of obtaining adequate MSNA data during PLM. However, it is not expected that PLM is a sympathoexcitatory maneuver and thus would not likely increase MSNA measures itself.

### Conclusion

In summary, heightened MSNA directly and negatively impacts the vasodilatory and hyperemic responses to PLM in young males. Additionally, PLM provides a unique approach to further understand the impact of heightened sympathetic activity on blood flow regulation without the confounding influence of local metabolic vasodilation and central command that occur with voluntary exercise. These findings add to our understanding of sympathetic control of the vasculature and underscore the importance of accounting for differences in sympathetic activity when examining vascular function by PLM.

## Data Availability

Data will be made available upon reasonable request.

## New & Noteworthy

Elevated sympathetic nervous system activity can impair vascular function. The impact of elevated muscle sympathetic nervous activity (MSNA) the passive leg movement (PLM) assessment of vascular endothelial function is not fully understood. We tested the hypothesis that increased MSNA, via post exercise circulatory occlusion (PECO), would blunt the PLM response. A progressive decline in PLM was observed with graded increases in MSNA indicating a direct, negative impact on the hyperemic and vasodilatory response to PLM.
